# Advancements and Challenges in Mouse Models for NK Cell-Based Cancer Immunotherapy

**DOI:** 10.3390/cancers18030384

**Published:** 2026-01-26

**Authors:** Chiara Vitale, Alessia Ruiba, Alessandra Dondero, Martina Serra, Alice Tassistro, Cristina Bottino, Roberta Castriconi

**Affiliations:** 1 Innate Pharma Research Laboratories, Innate Pharma, 13009 Marseille, France; chiara.vitale@innate-pharma.fr; 2 Clinical and Experimental Immunology UOC, IRCSS Istituto Giannina Gaslini, 16147 Genova, Italy; alessiaruiba@gaslini.org (A.R.); robertacastriconi@gaslini.org (R.C.); 3 Department of Experimental Medicine, Università di Genova, 16126 Genova, Italy; alessandra.dondero@unige.it (A.D.); martina.serra@unige.it (M.S.); tassistroalice@gmail.com (A.T.); 4 IRCCS Ospedale Policlinico San Martino, 16132 Genova, Italy

**Keywords:** natural killer cells, NK cell-based immunotherapy, humanized mouse models, cancer immunotherapy, CAR-NK cells, NK cell engagers, tumor microenvironment

## Abstract

NK cells are key components of the innate immune system, capable of recognizing and eliminating tumor or virus-infected cells and able to modulate both innate and adaptive immune responses. This makes NK cells attractive candidates for cancer immunotherapy, through passive approaches such as adoptive NK cell transfer, or active approaches aimed at enhancing endogenous NK cell activity in vivo. Promising results have emerged from preclinical studies and early-phase clinical trials. Nevertheless, the therapeutic efficacy of NK cell-based approaches is often limited by several factors, such as the poor NK cell persistence in vivo, the inefficient tumor infiltration, and the immunosuppressive milieu typical of the tumor microenvironment. The preclinical development of NK cell-based therapies relies largely on animal models. Humanized mouse models have evolved from early immunodeficient strains to more advanced systems incorporating human cytokines, which more effectively support NK cell development, maturation, and function. These models have substantially improved our understanding of human NK cell biology and enabled the evaluation of novel therapeutic strategies. However, further optimization is still required to better recapitulate the tissue-specific heterogeneity of human NK cells and their conditioning by the tumor microenvironment. In this review, we provide an overview of recent advances in the generation of humanized mouse models for NK cell-based cancer immunotherapy, discussing their advantages and limitations and highlighting how emerging technologies may contribute to the development of more predictive preclinical platforms.

## Simple Summary

Natural killer (NK) cells are innate immune cells with a critical role in the recognition and elimination of malignant cells and have therefore attracted considerable interest as targets for cancer immunotherapy. Despite encouraging results in experimental settings, the clinical performance of NK cell-based approaches can be improved. One of the major limitations is the inability of conventional animal models to accurately reproduce human NK cell development, persistence, and functional regulation within the tumor microenvironment. This review discusses the need for more advanced preclinical models and examines recent progress in the development of humanized mouse systems that more faithfully recapitulate key traits of human NK cells. By outlining both the advantages and the remaining limitations of these models, this work aims to inform the research community and support the design of more predictive preclinical studies, ultimately facilitating the translation of NK cell-based therapies into effective cancer treatments.

## 1. Introduction

Natural killer (NK) cells are cytotoxic members of the innate lymphoid cell (ILC) family, which play a central role in early defense against cancer and viral infections [1,2]. Upon recognition through a variety of activating receptors, NK cells eliminate transformed cells by releasing perforin- and granzyme-containing granules and through the activation of death receptor pathways, such as Fas/FasL, on transformed/infected cells. In addition to their cytolytic capacity, NK cells produce chemokines and cytokines, including IFN-γ, which shape and amplify both innate and adaptive immune responses [2]. NK cells originate from hematopoietic stem cells (HSCs) in the bone marrow (BM) and continue their maturation in secondary lymphoid tissues, following both linear and, as recently described, branched developmental pathways [3]. Moreover, NK cells include tissue-resident populations with distinct phenotypic and functional features, underlining the complexity of NK cell biology [4]. NK cell activity depends on a fine balance between inhibitory and activating receptors, which sense the presence of specific ligands on target cells [5]. Inhibitory receptors, such as Killer Ig-like Receptors (KIRs) and CD94/NKG2A, bind HLA class I molecules to ensure self-tolerance, driving NK cell “education”, and enabling competent effector functions [2,6,7,8]. Additional inhibitory pathways, including immune checkpoint receptors such as TIGIT, TIM-3, and PD-1, negatively regulate NK cell responses and are often exploited within the tumor microenvironment (TME) [8,9]. Conversely, activating receptors enable NK cells to recognize stress signals on transformed cells. Key activating receptors include natural cytotoxicity receptors (NCRs) (NKp30, NKp44, NKp46), NKG2D, activating KIRs, CD94/NKG2C, DNAM-1, and CD16A (FcγRIIIa), the latter mediating antibody-dependent cellular cytotoxicity (ADCC) [5,8,10]. The integration of these signals determines whether NK cells initiate cytotoxicity or cytokine production.

Human peripheral blood (PB) NK cells are classically divided into two major subsets: CD56^bright^ cells, which are potent cytokine producers with limited basal cytotoxicity, and the more mature and highly cytotoxic CD56^dim^ population, which expresses CD16A and higher perforin levels [11]. A third subset of tissue-resident NK cells is found in organs such as the BM, lymph nodes, spleen, and liver [12,13] and is characterized by CD69 expression, and phenotypic and functional traits distinct from circulating NK cells [12,13,14]. Another specialized population of adaptive or memory-like NK cells emerges following chronic stimulation, such as by CMV infection, and displays enhanced responsiveness upon reactivation [8,15]. Recent single-cell multi-omics approaches have further expanded the understanding of NK cell heterogeneity, revealing multiple NK cell states with distinct transcriptional programs, metabolic profiles, and developmental trajectories, challenging traditional classifications based solely on surface markers. These studies also show that the NK cell composition differs markedly across tissues and tumors, often being quite different from that observed in PB [3].

Within the TME, NK cell functions are tightly regulated by dynamic interactions with other immune cell populations [16]. Regulatory T cells (Tregs) suppress NK cell cytotoxicity and cytokine production through the release of immunosuppressive cytokines such as TGF-β and IL-10, as well as through metabolic competition and cell-to–cell contact-dependent mechanisms [17,18,19,20]. Myeloid-derived suppressor cells (MDSCs) also impair NK cell activity by producing reactive oxygen species, nitric oxide, arginase, and immunosuppressive cytokines, leading to reduced activating receptor expression and functional exhaustion [21,22,23]. Tumor-associated macrophages (TAMs), particularly those with an M2-like phenotype, contribute to NK cell dysfunction by secreting soluble mediators, including TGF-β and prostaglandin E2, and shaping an immunosuppressive milieu that limits NK cell infiltration and effector function [24,25]. Conversely, appropriately activated NK cells can counteract the immunosuppressive circuits within the TME by producing immunostimulatory cytokines such as IFN-γ, which also promotes a switch in the macrophage polarization toward a pro-inflammatory phenotype [26].

NK cells’ ability to eliminate malignant cells, combined with a favorable safety profile and minimal risk of graft-versus-host disease (GvHD) compared with T cell-based therapies [27,28,29], makes them strong candidates for both adoptive transfer and in vivo engagement strategies. Despite significant progress, the clinical translation of NK cell-based therapies, especially for solid tumors, still requires substantial optimization. A key obstacle is the difficulty of accurately modeling NK cell behavior in preclinical settings, including their persistence and trafficking in vivo, and interactions with human immune and stromal compartments. NK cell-based immunotherapies include a broad range of strategies, from adoptive passive approaches to active engagement modalities, and their fast validation depends on the availability of robust preclinical platforms. In this context, humanized mouse models have gained increasing attention. This review outlines the development, strengths, and limitations of current humanized mouse platforms and discusses how their refinement could improve their ability to anticipate clinical outcomes.

## 2. NK Cell-Based Immunotherapies in Oncology: Approaches, Mechanisms, and Challenges

NK cell ability to combine natural cytotoxicity with cytokine-mediated modulation of innate and adaptive immunity has made NK cells highly attractive tools in cancer immunotherapy [30,31,32,33]. Over the past decade, NK cell-based therapies have progressed from fundamental experimental approaches to clinically actionable platforms and can be broadly categorized into passive and active approaches [34]. Passive strategies involve the adoptive transfer into patients of in vitro cytokine-expanded and/or genetically engineered NK cells. These therapies use NK cells from healthy donors (HDs) or from cancer patients, providing recipient patients with a population of highly cytotoxic and viable NK cells [35,36]. Active strategies, on the other hand, are mainly aimed at harnessing and potentiating the patient’s own endogenous NK cells, and can include the administration of molecular engagers such as monoclonal antibodies (mAbs) [37], bispecific (BiKEs), or tri-specific (TriKEs) NK cell engagers [38,39,40], and the recently described tri- or tetra-specific Antibody-based NK cell Engager Therapeutics (ANKETs) [41,42,43]. All these tools are designed to be administered to patients, facilitating in vivo NK cell activation and tumor recognition.

The distinction between passive and active strategies reflects key differences in mechanistic approach, scalability, and translational challenges. Passive therapies allow us to precisely define the NK cell product in terms of cell number, phenotype, grade of activation, and genetic modifications, supporting the creation of tailored products. Some examples of genetic manipulation are Chimeric Antigen Receptor (CAR)-NK [44,45] or induced pluripotent stem cell (iPSC)-derived NK cells [46]. On the contrary, active strategies exploit existing NK pools, avoiding the complexities of cell manufacturing and potentially promoting more durable in vivo responses. Despite differences, both approaches share similar hurdles, such as the NK cell trafficking to tumor sites and the immunosuppression occurring in the TME [47]. Understanding the strengths and limitations of passive and active strategies is therefore critical for designing efficient patient treatments, and innovative preclinical models are necessary to analyze the efficacy and compare different therapeutic strategies.

### 2.1. Passive Strategies: Adoptive NK Cell Transfer

**Figure 1:**
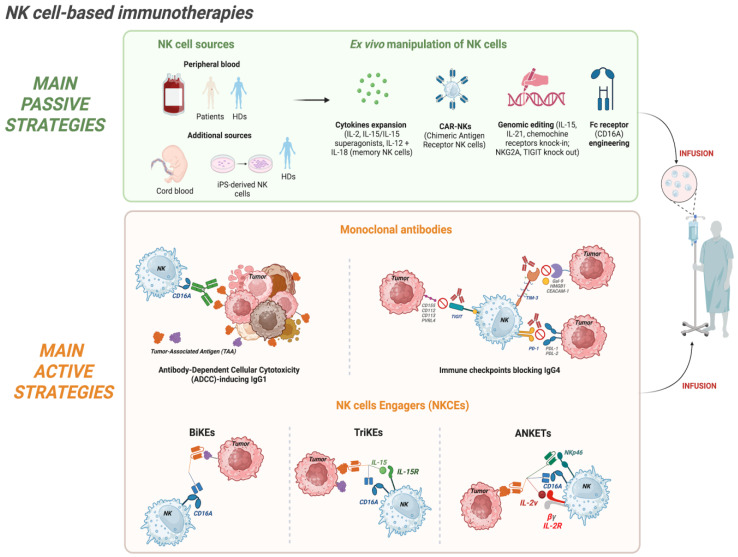
Summary of the main passive and active NK cell-based immunotherapeutic strategies. Created in https://BioRender.com.

For passive strategies, the main challenge is certainly to obtain an adequate NK cell product in terms of number, functionality, and in vivo persistence [48,49,50]. Thus, technological advances, including optimized cytokine-based expansion protocols [51,52], feeder-cell co-culture systems, and gene engineering [53], are being used to transform NK cells into a viable therapeutic platform (Figure 1). Multiple sources of NK cells are now employed: the most common source is represented by the PB of patients or HDs, but additional sources are umbilical cord blood (UBC), BM, and human embryonic stem cells (hESCs) or iPSCs [54,55,56]. Additionally, the NK cell product can be represented by immortalized NK cell lines such as NK-92 [57]. iPSC-derived NK and NK cell lines in particular offer unlimited scalability, standardized phenotypes, and the possibility of complex genetic modifications, thus representing a promising tool for generating “off-the-shelf” therapies that can be consistently manufactured with defined functional properties [58]. Ex vivo cytokine stimulation remains central in adoptive NK cell therapy. The cytokines used span from the seminal interleukin-2 (IL-2) [59] to IL-15 or IL-21, which play key roles in NK cell differentiation, proliferation, survival, and activation [60,61,62]. Special attention was paid to IL-15 due to the reduced toxicity as compared to IL-2 and its ability to support NK persistence without expanding regulatory T cells. Along this line, IL-15 super-agonists (for example, N-803/ALT-803) have been developed [63]. Interestingly, combinations of IL-12 and IL-18 can generate cytokine-induced memory-like NK cells, which exhibit enhanced cytotoxicity and prolonged activity in vivo [64,65]. Nonetheless, in clinical and preclinical studies, in vivo long-term persistence beyond two to three weeks remains uncommon [66,67], and trafficking into solid tumor sites is frequently inefficient [68], highlighting an important weakness in current passive NK cell-based therapies.

Genetic engineering has enabled a new generation of adoptive NK cell-based therapies, most notably the infusion of CAR-NK cells. These products combine the innate antigen-specific recognition and cytotoxic potential of NK cells with an additive mechanism represented by the expression of an engineered tumor-targeting chimeric receptor [28,69]. For instance, adult PBMCs and UBC-derived CD19 CAR-NK cells have demonstrated promising clinical efficacy in hematologic malignancies, achieving durable responses without severe cytokine release syndrome or neurotoxicity [70,71]. Further engineering strategies aim to enhance CAR-NK cell persistence, improve tumor homing, and overcome inhibitory pathways. In this context, it was shown that the co-expression of IL-15 [72] or IL-21 promotes CAR-NK expansion and survival [73].

Beyond CAR-based approaches, additional genetic engineering strategies are being explored to enhance NK cell efficacy. For example, the introduction of a gene coding for chemokine receptors (e.g., CXCR4, CCR7, or CCL21) facilitates trafficking to tumor sites [74,75,76]. Moreover, the CRISPR/Cas9-mediated knockout of inhibitory receptors such as NKG2A or TIGIT is also being explored [77,78,79].

Finally, iPSC-derived NK cells represent an especially versatile platform, allowing for a scalable and standardized “off-the-shelf” product [46,80]. Unlike primary NK cells, which show a huge donor-related heterogeneity and limited expansion capacity, homogeneous iPSC-NK cell products can be obtained, addressing the reproducibility of the results and enabling complex genetic engineering. In recent years, several studies have highlighted the potential of these cells in advanced preclinical models. For example, CRISPR/Cas9-mediated deletion of the CISH protein generated iPSC-NK cells with enhanced JAK-STAT signaling and improved proliferation under low cytokine conditions, along with in vivo persistence, metabolic fitness, and resistance to functional exhaustion [81]. Similarly, overexpression of CD226 enhanced cytotoxicity against acute myeloid leukemia (AML) cells, demonstrating how targeted activation receptor modifications can improve the antitumor efficacy [82]. Another innovative approach involved engineering strategies to obtain a high-affinity Fc receptor, such as that derived from the CD64/CD16A fusion. The expression of this construct, integrated with IL-15 coding modules, allowed iPSC-NK cells to be preloaded with tumor antigen-specific mAbs for flexible targeting of multiple tumors, including lymphoma. Importantly, iPSC-derived NK cells expressing CD64/16 FcγR showed cytotoxic activity preserved after cryopreservation [83].

More recently, engineering strategies have combined CCL19, CCR2B, NKG2D, high-affinity CD16A, and IL-15 into a single iPSC-NK line, resulting in cells with improved tumor infiltration and enhanced antitumor activity in solid tumor models [84]. These studies underscore how the iPSC-NK platform represents a rational, flexible engineering strategy, paving the way for advanced pre-manufactured NK cell therapies in cancer immunotherapy. However, limitations on the use in vivo of iPSC-NK are present, including incomplete terminal maturation with reduced cytotoxicity and cytokine production, still poorly characterized differences among different iPSC clones, and their potential dangerousness due to genomic instability with the risk to acquire chromosomal abnormalities or mutations during reprogramming and in vitro expansion [58,80]. Moreover, iPSC-based strategies require complex manufacturing and high costs.

Concerning the NK cell differentiation, it is worth noting that in vivo, the physiological process requires key cell-to-cell signals provided by stromal cells. In their absence, the NK cell maturation trajectory and/or their functional programming may be distorted. Future refinement of iPSC differentiation protocols, incorporation of human stromal co-culture systems, and engineering of more physiologic cytokine niches within humanized mice will be fundamental to improving the translational efficacy of iPSC-NK platforms.

### 2.2. Active Strategies: NK Cell Engagers (NKCEs)

While passive therapies deliver an in vitro optimized NK cell product, active strategies aim to potentiate/expand endogenous NK populations in vivo through targeted molecular engagement. These approaches include chimeric or humanized mAbs that block inhibitory axes such as immune checkpoints (IgG4 mAbs) or trigger ADCC (IgG1 mAbs), as well as engineered tools such as BiKEs, TriKEs, and ANKETs, which engage multiple NK receptors and tumor antigens (Figure 1). Active strategies are very appealing because they do not require specialized infrastructure for cell manipulation. Together with their scalability and their potential to engage NK cells across multiple tissue compartments, they are among the therapeutic strategies with the broadest applicability to date.

mAbs such as rituximab (anti-CD20), trastuzumab (anti-HER2), and cetuximab (anti-EGFR) induce ADCC by engaging NK cells expressing CD16A, which bind the Fc region of IgG antibodies, thereby triggering degranulation and tumor cell lysis [85,86]. Next-generation Fc-engineered antibodies, such as margetuximab (anti-HER2) [87], or afucosylated antibodies, such as obinutuzumab (anti-CD20) [88], have been shown to have good clinical efficacy by enhancing NK cell activation in both hematological and solid tumors [89,90,91]. Moreover, the association of these ADCC-optimized antibodies with pro-inflammatory cytokines (e.g., IL-15 superagonists) [63,92,93] or checkpoint blockade (e.g., anti-NKG2A [94]) further boosts their efficacy. However, it must be considered that CD16A expression is not a phenotypic trait of all human NK cells. In fact, while approximately 90% of blood NK cells in adult HDs expressed CD16A, in other tissues, its expression is confined to a variable percentage of cells, which in some cases, could be very low [3]. This prompted researchers to develop bi/tri/tetra-specific NK cell engagers able to overcome this limitation.

With these premises, several groups have started investigating NKCEs in solid cancers, using their modular structure to combine the engagement of NK-activating receptors with the recognition of tumor-associated antigens (TAAs) in epithelial malignancies [42]. Although still early, these studies underscore the versatility of NKCEs and their potential to extend NK cell-based immunotherapy beyond onco-hematological disease.

These multi-specific molecules are engineered tools combining different single-chain variable fragment (scFv) portions of mAbs specific for different NK activating receptors (such as CD16A, NKp30, NKp46, NKG2D) and TAAs, forming a functional effector/target bridge that activates NK cell cytotoxicity [42,43,95,96,97]. Preclinical studies of CD16A × CD33 [39,98] or CD16A × CD123 BiKEs [99] demonstrated effective activation of NK cells against primary leukemic and myelodysplastic stem and progenitor cells, highlighting their potential as immunotherapeutic strategies for AML, myelodysplastic syndrome, and acute lymphoblastic leukemia (ALL). In a separate study, dual targeting of glioblastoma cells using bispecific killer cell engagers directed at EGFR and HER2 enabled efficient killing by NKG2D-CAR-engineered NK cells [100]. Recent work has expanded the specificity of NKCEs, providing proof of concept that multi-specific molecules can be tailored toward diverse TAAs and employed across different oncological settings. TriKEs frequently incorporate an IL-15 moiety, sustaining NK proliferation and cytotoxic function during engagement [101,102]. TriKEs have been shown to induce higher IFN-γ production than BiKEs, indicating stronger NK cell activation and tumor control in preclinical mouse models. Importantly, the degree of cytokine release remains within a favorable safety profile. In pediatric B-cell precursor-ALL, NKCEs triggering either NKp46 or NKp30, in combination with CD16A, and targeting CD19 or CD20 on tumor cells, demonstrated potent enhancement of NK cell degranulation and IFN-γ production [103]. More recently, Gauthier et al. reported a trifunctional NKCE targeting CD123 in AML and co-engaging NKp46 and CD16A on NK cells [97]. This NKCE was shown to overcome CD64-mediated resistance: the high-affinity IgG receptor CD64 sequesters conventional anti-CD123 IgG1 mAbs, rendering them effective only against CD64^−^ target cells. In contrast, the CD123-NKCE retained its activity regardless of CD64 expression. Moreover, it induced NK cell activation only in the presence of AML cells and maintained a safe inflammatory and cytokine release profile. In fact, in xenogeneic and non-human primate studies, it provided enhanced disease control, prolonged depletion of CD123^+^ cells, and no detectable toxicity, supporting its progression toward clinical development [97].

Novel anti-CD20 tetrameric NK cell engagers (ANKETs) have recently been validated in preclinical models. These molecules are designed to selectively co-engage on NK cells NKp46 and CD16A through an Fc portion, and the IL-2Rβγ chain through an IL-2 variant (IL-2v), while simultaneously targeting CD20, to enable directed cytotoxicity. IL-2v carrying a point mutation does not bind to the IL-2Rα (CD25) chain, constitutively expressed by Tregs and by other activated cells, including endothelial cells, thus limiting Treg expansion and other adverse effects such as pulmonary edema [104,105]. aCD20/aNKp46/Fc/IL2v tetra-specific ANKETs activate and expand NK cells in vitro and promote their accumulation within tumors in vivo, resulting in superior tumor control in aggressive lymphoma mouse models compared with conventional antibodies such as the anti-CD20 obinutuzumab. Importantly, in non-human primates, the molecule induced B-cell depletion with only minimal and transient cytokine release, indicating a favorable safety profile [43]. Based on that, Demaria et al. focused on the detailed characterization of IPH6501, a clinical-grade aCD20/aNKp46/Fc/IL2v ANKET developed for the treatment of B-cell non-Hodgkin lymphoma (B-NHL) [106]. This study reinforced the earlier results by demonstrating superior cytotoxic activity in vitro compared with both therapeutic anti-CD20 IgG1 antibodies (rituximab and obinutuzumab) and CD20-specific T-cell engagers (TCEs). Moreover, at comparable functional doses, IPH6501 induced lower and transient levels of pro-inflammatory cytokines (TNF-α and IL-6) than CD20-TCEs, suggesting a reduced risk of cytokine release syndrome (CRS). In addition, single-cell RNA-seq (scRNA-seq) demonstrated that the treatment activates all major NK cell subsets, favoring the emergence of cytokine-induced memory-like (CIML) NK cells, known for potent antitumor activity and persistence. Finally, IPH6501 induced an antigen-independent antitumor activity by upregulating the expression of key activating receptors such as NKG2D. This effect may be beneficial, as it could exploit the natural NK cytotoxicity, helping to counteract the survival and expansion of CD20-negative tumor variants that commonly emerge in patients following CD20-targeted immunotherapies [106].

Collectively, these results highlight ANKETs as versatile next-generation immunotherapeutic agents capable of harnessing NK cells for potent yet safe antitumor responses. NKCEs show a favorable safety profile also because NK cells produce fewer pro-inflammatory cytokines than T cells and are mainly activated in the presence of target tumor cells. Despite these advantages, both passive and active NK cell-based strategies face key limitations: limited NK cell persistence and trafficking in vivo, manufacturing complexity and immunogenicity risks for passive approaches, and reduced responsiveness of patient NK cells or their suppression in the TME for active ones. The limited clinical evidence, particularly in solid tumors, calls for caution and underscores the need for preclinical models that better recapitulate human NK cells and the TME [107,108,109,110]. The following chapters provide an overview of efforts addressing these challenges.

## 3. Preclinical Humanized Mouse Models for Assessing NK Cell-Based Strategies

### 3.1. Early Immunodeficient Models

**Figure 2:**
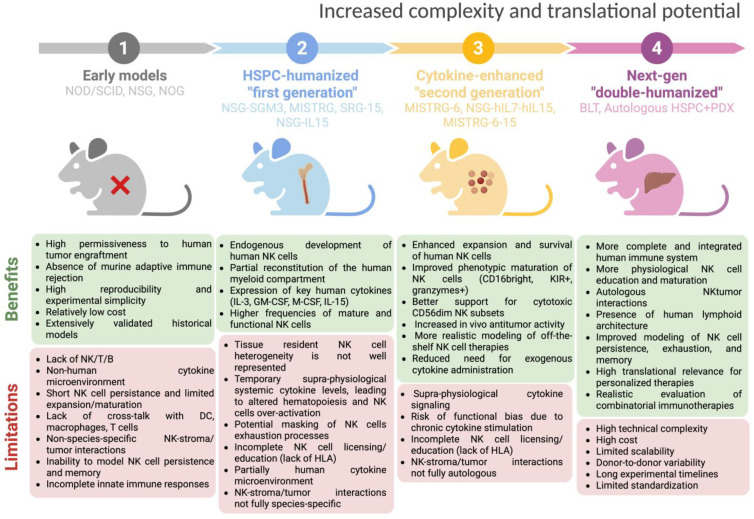
Progressive evolution of humanized mouse models for studying human NK cell biology and antitumor functions. Created in https://BioRender.com.

The seminal approaches for the preclinical evaluation of NK cell therapy through animal models comprised immunodeficient strains such as Non-Obese Diabetic/Severe Combined Immunodeficient (NOD/SCID) mouse [111], NOD/SCID/γc^null^ (NSG) mouse [112], and NOG mouse (NOD/Shi-scid/IL2Rγ^null^) [113], which lack functional T, B, and NK cell compartments (by virtue of IL2Rγ knockout) (Figure 2). These mice allowed the xenotransplantation of human tumor cells, including those patient-derived (PDX models), and intravenous injection of human immune effectors, including NK cells from HDs or engineered NK cell lines, enabling the first evaluation of human tumor engraftment and the NK cell-mediated antitumor activity [114,115,116,117,118].

Nevertheless, these models exhibited major translational gaps. One key limitation is the poor interspecies cross-reactivity, along with the lack of homologous cytokines, such as IL-15 and IL-21, able to efficiently sustain human NK cell differentiation and activation. Consequently, human NK cells engrafted in such hosts are short-lived and exhibit limited expansion or functional maturation [119,120]. In addition to that, the human stromal and vascular microenvironment is progressively replaced by host-derived murine stroma, which differ from human counterparts in extracellular matrix (ECM) composition and signaling networks. These differences result in non-identical chemokine gradients and signaling networks compared with human tumors, potentially influencing immune cell trafficking cues, including those relevant for NK cell recruitment and function [121,122,123,124,125,126]. Furthermore, in vitro documented crosstalk between human NK cells and other immune lineages, such as dendritic cells, macrophages, and T cells, cannot be modeled in these animal models.

### 3.2. Progress Towards the First Humanized Immunodeficient Models to Study Human NK Cells

To address some of the above-mentioned issues, the field has advanced to generate humanized mouse models in which human hematopoietic stem cells and progenitor cells (HSPCs, CD34^+^) are engrafted into immunodeficient recipients, thereby generating a full human immune system within the murine host. These HSPC-engrafted humanized systems are more suitable for investigating not only the NK cell antitumor activity but also NK cell ontogeny, differentiation, trafficking, and infiltration into human xenografted tumors.

The most widely used humanized mouse models are NSG-SGM3 [127], MISTRG [120], SRG-15 [128], and NSG-IL15 models [129]. Specifically, NSG-SGM3 mice are based on the highly immunodeficient NSG background and are transgenic for human SCF, GM-CSF, and IL-3; these cytokines enhance the development of human myeloid lineages, which in turn support NK cell maturation and function, although the endogenous levels of human IL-15 are still scarce [127]. MISTRG mice (M-CSF^h/h^ IL-3/GM-CSF^h/h^ SIRPα^h/h^ TPO^h/h^ RAG2^−/−^ IL2Rγ^−/−^) incorporate knock-ins of human M-CSF, IL-3/GM-CSF, TPO (thrombopoietin), and SIRPα in mice deficient for T/B and NK cells, enabling efficient development not only of human myeloid cells but also of NK cells that display maturation, cytotoxicity, and tissue distribution more closely resembling that in humans [120]. In this model, the human engraftment is favored by the presence of transgenic human SIRPα, which provides a “don’t eat me signal” reducing the phagocytosis of human stem cells and developing an immune compartment.

In SRG-15 mice with a RAG2^−/−^ IL2Rγ^−/−^ background, the gene coding for the murine IL-15 is replaced with the human counterpart, along with a knock-in of the human SIRPα. This results in a physiological expression of IL-15 in a tissue- and cell-specific manner, which results in robust populations of human NK cells trafficking and infiltrating xenografted tumors and interacting with human T cells, dendritic cells, and macrophages [128]. In particular, in SRG-15 mice, de novo-derived human NK cells have been shown to infiltrate lymphoma xenografts and mediate ADCC following rituximab treatment.

Finally, in NSG-IL15 mice, human IL-15 is constitutively expressed (via transgenic or knock-in design), and provides particularly robust human NK cell reconstitution: high frequencies of mature and functional NK cells across multiple tissues, support for long-term survival without inducing GvHD, and enabling the physiologically relevant NK-mediated control of both tumors and infections [129,130,131]. Experiments on patient-derived melanoma in NSG-IL15 mice showed slower tumor growth compared to NSG mice, indicating that the enhanced NK cell compartment confers a significantly improved tumor-control effect [130].

Thus, growing evidence points to the superior reliability of humanized mouse models for studying human NK cell behavior in cancer, as compared with first-generation standard models such as NOD/SCID or NSG mice.

### 3.3. Tumor Type-Specific Considerations in Preclinical NK Cell Studies

Although humanized mouse models are increasingly used to evaluate NK cell-based immunotherapies, the selection of these models and the interpretation of their readouts critically depend on the tumor type. Across different oncological contexts, distinct biological and technical challenges should be carefully considered when selecting and interpreting preclinical models. In particular, experimental requirements may substantially differ between hematologic malignancies, solid tumors, and brain tumors.

In hematologic malignancies, tumor cells are typically localized in the PB, BM, or secondary lymphoid organs, making them more accessible to circulating NK cells [68,132]. As a result, intravenous treatments in immunodeficient or humanized xenograft mice are particularly informative for evaluating NK cell cytotoxicity, in vivo persistence, expansion, and ADCC. These models have been used for the preclinical validation of adoptive NK cell transfer (e.g., [133]), CAR-NK cells (e.g., [72,134,135]), and NKCEs (e.g., [43,97,99]). Humanized mouse strains supporting robust NK cell reconstitution, such as NSG-IL15 or SRG-15, further improve the predictive value of these studies by enabling sustained NK cell survival and functional maturation, as already discussed above.

Solid tumors represent a more complex experimental setting, in which therapeutic efficacy depends not only on intrinsic NK cell cytotoxicity but also on efficient trafficking, infiltration, and functional adaptation within a TME that can be highly immunosuppressive. Physical barriers such as a dense extracellular matrix, abnormal vasculature, and stromal components limit immune cell penetration, while local cytokines, metabolic constraints, and inhibitory ligands actively suppress NK cell activity [136,137,138]. Consequently, subcutaneous or orthotopic solid tumor models in humanized mice are extremely important to assess NK cell homing, persistence within tumor tissues, and susceptibility to TME-mediated dysfunction [118,139]. In this context, cytokine-enhanced humanized models (e.g., MISTRG, MISTRG-6-15, or IL-15 knock-in strains) provide a clear advantage over first-generation immunodeficient mice by partially restoring NK cell development, tumor infiltration, and crosstalk with other immune compartments [130,140]. For example, enhanced development and functional maturation of human NK cells in NSG-Tg (Hu-IL15) humanized mice have been shown to limit the growth of solid tumor xenografts such as melanoma PDX, indicating that humanized models can support antitumor NK responses in vivo [130]. However, limitations in stromal humanization and tissue-resident NK cell differentiation still constrain the faithful modeling of human solid tumors.

Brain tumors constitute a further distinct scenario, being characterized by unique anatomical and immunological constraints. The blood–brain barrier (BBB) severely limits immune cell trafficking into the central nervous system in physiological conditions via specialized tight junctions and unique endothelial properties [141,142]. The brain microenvironment is populated by specialized resident cells, including microglia and astrocytes, which play central roles in shaping immunity and tumor progression; these glial elements are distinct from conventional stromal fibroblasts found in peripheral tumors, and microglia often form a major fraction of TAMs in gliomas [143,144,145]. NK cell infiltration into brain tumors is typically low, and cues regulating NK cell activation and persistence differ markedly from those in peripheral tissues [146,147,148]. As a consequence, orthotopic brain tumor models are used to evaluate NK cell-based therapies. For example, preclinical glioblastoma (GBM) research critically relies on orthotopic mouse models, as they recapitulate key histopathological hallmarks of the human disease, including dense vascularization, and a pseudopalisading, highly invasive growth, with cells arranging themselves in fence-like rows (palisades) around areas of necrosis. Compared with ectopic (subcutaneous) xenografts, orthotopic models enable a more realistic evaluation of the therapeutic efficacy, overall survival, and ability of treatments to overcome the BBB. However, still major limitations exist, including interspecies differences in brain architecture and genetics, the need to use immunodeficient hosts for human xenografts, and the genetic drift associated with long-term cultured cell lines [149,150,151,152,153,154].

Experimental systems specifically designed to evaluate NK cell-based immunotherapies in intracranial tumors remain numerically limited and technically demanding. Only a restricted number of studies have employed orthotopic glioma models to directly investigate NK cell trafficking, persistence, and antitumor activity within the central nervous system, often relying on stereotactic implantation and local or intratumoral administration to bypass the constraints imposed by the BBB. Early preclinical studies demonstrated that intracranial injection of IL-2-activated NK cells could markedly enhance the efficacy of fixed tumor vaccines, inducing significant tumor regression in settings where vaccination alone was ineffective [155]. However, more recent investigations in immunocompetent orthotopic glioma models have highlighted persistent limitations of NK cell-based approaches in vivo, including reduced persistence and an inferior therapeutic efficacy compared with CAR-T cells or combinatorial strategies involving CAR-T and CAR-NKT cells [156]. Despite these challenges, accumulating evidence indicates that intrinsic limitations of NK cells may be partially overcome through genetic engineering approaches, such as the co-expression of pro-inflammatory cytokines (e.g., IL-12 or IFN-α2), which enhance NK cell survival, functional persistence, and antitumor activity within the highly immunosuppressive brain tumor microenvironment [157]. Together, these observations highlight the need for more refined and standardized models to accurately assess NK cell-based immunotherapies in the brain. Overall, current evidence indicates that future progress will depend on the development and implementation of more faithful and complex platforms, including PDX, advanced humanized mouse models, and three-dimensional organoid systems, which more closely reflect the genetic and immunological landscape of human GBM [158,159,160].

Overall, these differences highlight the absence of a universally applicable preclinical model for all cancer types. Instead, the choice of experimental system should be guided by the specific biological questions being addressed, such as cytotoxic potency, persistence, trafficking, resistance to immunosuppression, and the anatomical and microenvironmental features of the tumor under investigation. The tumor type-specific requirements are essential to improve the translational relevance of preclinical studies evaluating NK cell-based immunotherapies, providing an essential framework for interpreting the strengths and limitations of humanized mouse models.

## 4. Burning Challenges and Limitations of Current Humanized Mouse Models for Studying NK Cell-Based Therapies

Despite the described progress in the establishment of preclinical humanized mouse models, significant limitations remain, primarily due to inter-species differences in immune system composition, cytokine signaling, tissue microenvironments, and NK cell biology. These discrepancies substantially affect the interpretation and the translation of preclinical findings, limiting the accurate prediction of the immunotherapies’ benefits [161,162,163]. In healthy humans, PB-NK cells constitute approximately 5–15% of circulating lymphocytes, with a major proportion (~90%) of highly cytotoxic CD56^dim^ cells. A minor fraction (~10%) is represented by poor cytotoxic and cytokine-secreting CD56^bright^ cells [164,165]. As previously mentioned, CD56^dim^ NK cells mediate direct cytotoxicity via perforin and granzymes, whereas CD56^bright^ NK cells mainly orchestrate innate and adaptive immune responses through cytokine and chemokine secretion, influencing dendritic cell (DC) and macrophage [166] maturation and T-cell priming [2,167].

In most humanized mouse models, the peripheral NK compartment is not only consistently under-represented but also fails to mirror the human distribution between the two main subsets [118,130,168]. For example, NSG mice engrafted with human CD34^+^ HSCs typically show a predominance of CD56^bright^ NK cells in the peripheral blood, whereas the CD56^dim^/CD16^+^ subset is virtually absent. On the contrary, the more complex NSG-Tg (Hu-IL-15) model [130] supports the development of both CD56^bright^ and CD56^dim^ NK cell compartments, resulting in a significantly improved representation of circulating human NK cells. Although this model does not fully recapitulate the human NK cell scenario, it more closely resembles the relative proportions of the major circulating NK cell subsets observed in humans. Moreover, NSG-Tg (Hu-IL-15) mice showed higher levels of functional granzyme A/B^+^, perforin^+^ NK cells in both PB and the spleen compared to NSG model. Even more advanced models such as the previously described MISTRG model, despite representing a significant improvement with respect to first-generation humanized models in supporting human NK cell development, NK cell maturation, tissue distribution, and functional diversity, remain incomplete and highly dependent on additional human cytokine support. Thus, nowadays, we are far from having a mouse model that fully recapitulates the human NK cell compartment [119,128,169].

In fact, human NK cells exhibit a huge tissue heterogeneity, with specific subsets residing in secondary lymphoid organs and peripheral tissues [3,5,170,171,172]. For instance, liver-resident CD56^bright^CD69^+^CXCR6^+^ NK cells display low cytotoxicity but a high cytokine output, contributing to immune surveillance and tolerance [12,171,173,174]. Lung NK cells acquire adaptive-like, memory features following viral exposure [175,176], whereas uterine NK cells play critical roles in vascular remodeling during pregnancy [177,178]. Lymphoid tissue NK cells regulate DC maturation and T-cell priming, demonstrating the interplay between NK cells and adaptive immunity [179,180,181].

Conventional humanized mouse models fail to faithfully reproduce this tissue-specific heterogeneity because the signals required for tissue-resident NK differentiation, such as local cytokines, adhesion molecules, and stromal cues, are largely absent due to the lack of interspecies cross-reaction [121,123,124,125]. In fact, in humanized mice, multiple species-specific mismatches exist. Stromal and tissue-resident immune compartments remain predominantly murine. For instance, murine IL-15 poorly binds human receptors, leading to incomplete NK maturation, reduced survival, and impaired cytotoxicity [130,182]. Consequently, humanized mice harbor PB-like CD56^dim^ NK cells in the circulation but virtually lack tissue-resident NK phenotypes. These data indicate that conventional humanized mice inadequately model organ-specific NK cell properties, severely limiting studies on solid tumors where tissue-resident NK cells are critical for controlling tumor growth and metastasis.

Cytokine-humanized mouse models improved human NK cell development. However, the various strategies used to introduce human cytokines can generate expression patterns that deviate from physiological conditions, sometimes resulting in temporarily abnormally higher systemic cytokine levels. This can lead to altered hematopoiesis and over-activation of NK cells, which also present defects in maturation and licensing [125,183,184]. Thus, cytokine-engineered models only partially correct for NK developmental defects, and the cytokine environments they establish may influence NK homeostasis, potentially obscuring processes such as exhaustion or the switch on of negative regulatory programs.

As previously noted, cross-species differences in the chemokine compartment represent another limiting factor. Mice and humans differ in the presence and regulation of chemokines (e.g., absence of CXCL8 in mice, differential CXCL9/10/11 and CCL5 signaling), leading to a non-physiological NK recruitment, retention, and organ-specific trafficking [123,124]. Importantly, the human NK cell “education” or “licensing”, a process that leads to a progressive acquisition of the cytolytic machinery, relies on interactions of NK cells with autologous HLA class I^+^ DCs and macrophages. This process is poorly recapitulated in the presence of murine myeloid cells, leading to incomplete licensing, altered receptor expression, and reduced secretion of IFN-γ and cytotoxic granules [168,183,185,186]. In many humanized tumor models, the human stromal compartment is progressively replaced by murine stromal cells (fibroblasts, ECM, vasculature, other stromal elements), thereby altering the cellular and molecular composition of the TME [187,188,189]. Given species-specific differences in stroma-derived signals (e.g., cytokines, growth factors), this replacement may compromise the fidelity of cancer–stroma and immune–stroma interactions present in the human TME. Even if direct experimental data comparing oxygen, nutrient, or metabolite gradients between human tissues and murine-stromal xenografts remain unexplored, it can be hypothesized that such stromal replacement could produce altered metabolic landscapes with potential consequences for the metabolic adaptation, survival, trafficking, and effector function of human NK cells engrafted in humanized mouse models. Therefore, when interpreting results from NK cell-based immunotherapy studies in in vivo models, the impact of stromal replacement should be carefully considered. Most xenografted humanized mouse systems lack human antigen expression on normal murine tissues [163,190]. Tissue-humanized mouse models, such as the humanized liver, have been developed; however, they remain technically challenging and often preserve murine tissue/metabolism, limiting their utility for informative organ-specific toxicity studies. Simultaneous humanization of hematopoietic and non-hematopoietic tissues remains largely incomplete [191]. The limited reconstitution and the poor tumor infiltration of functional human NK cells in conventional mouse models [139], together with the progressive replacement of human stromal components by murine cells in humanized models [125], have historically hampered the effective evaluation of the negative impact of the TME-mediated immunosuppression in patients. In fact, phenotypic and functional profiling of tumor-resident NK cells in humans has clearly demonstrated the presence of exhausted cells, expressing high levels of inhibitory immune checkpoint receptors (e.g., PD-1, TIGIT, TIM3), downregulation of activators (e.g., NKp30, NKG2D, and DNAM-1), and enrichment in the CD16A^−^ population [192,193,194,195,196]. Thus, ideally, only the reproduction of these NK cell properties in the preclinical mouse model could allow a reasonable evaluation of therapies’ efficacy.

In this context, MISTRG-6-15 mice with knocked-in human IL-6 and IL-15 provide robust support for the expansion of IL-15-armored or CAR-engineered NK cells and have been used to study NK cell exhaustion in the settings of chronic infections such as HIV. This study showed that NK cells significantly contribute to anti-HIV-1 responses in vivo, but this activity was reduced in lymphoid organs, suggesting a tissue-specific responsiveness of NK cells to certain therapies [197]. So far, published studies specifically evaluating NK cell exhaustion in tumor-bearing MISTRG-6-15 mice are still absent.

Recent evidence indicates that sustained CAR-NK activation, driven by continuous CAR signaling through the CD3ζ domain or by elevated levels of IL-15, upregulates the transcription factor CREM, which functions as a negative regulator. CREM induction is primarily mediated by the PKA–CREB axis, activated downstream of ITAM-dependent CAR signaling as well as IL-15 stimulation. CREM drives epigenetic and transcriptional reprogramming of CAR-NK cells, repressing key effector genes such as GZMB and IFNG while promoting exhaustion- and stress-associated programs. Together with that, engineered CAR-NK cells co-expressing soluble IL-15 have been linked to severe toxicity in animal models, as systemic accumulation of IL-15 can trigger uncontrolled CAR-NK expansion. Safer strategies such as membrane-bound IL-15 provide spatially restricted and more physiological cytokine signaling: this enhances the efficacy and persistence of NK cells while maintaining a favorable safety profile, avoiding uncontrolled proliferation, acute toxicity, and organ damage associated with soluble IL-15 [198,199,200].

In summary, current humanized mouse models, although providing significant help for the study of NK cell-based immunotherapies, still demonstrate critical limitations in mirroring human NK biology. This underscores the need for next-generation approaches, including multiple knock-in strategies, organoid integration, iPSC-derived NK cells, and CRISPR-based tissue humanization, to enhance NK maturation, preserve tissue heterogeneity, enable functional plasticity, and improve the translational relevance of preclinical studies.

## 5. Next-Generation Humanized Mouse Models and Innovative Technologies Unraveling Human NK Cell Dynamics in Tumor Immunity

As mentioned above, NSG mice expressing transgenic human IL-15 exhibit an improved development, survival, and in vivo function of NK cells [129,130,131]. Nevertheless, it was demonstrated that constitutive IL-15 expression could drive supra-physiological NK cell expansion, which can obscure emerging exhaustion and checkpoint regulation, and which has also been shown to promote functional NK cell dysfunction [201].

To address these potential drawbacks, and recognizing that insufficient human cytokine support was a major bottleneck for human NK cell development in vivo, more recent cytokine-enhanced humanized strains have incorporated additional human cytokines to further improve NK cell robustness. Beyond the strains already described in Section 2.2, expanded cytokine knock-in models such as NSG-hIL7-hIL15 [202] and MISTRG6 (expressing human M-CSF, IL-3, GM-CSF, SIRPα, TPO, and IL-6) [203] have demonstrated even greater improvements in human NK cell development and survival. Matsuda et al. showed that NSG-hIL7-hIL15 mice engrafted with human CD34^+^ HSPCs generate extremely high NK cell frequencies, approximately 43% of human CD45^+^ cells in PB, far exceeding earlier models [202]. A particularly compelling study used MISTRG6 mice engrafted with patient-derived BM HSPCs, followed by implantation of a matched PDX tumor. This strategy created a fully autologous, genetically matched humanized model for the prospective study of tumor–immune interactions in patients with solid tumors. Humanization of the IL-6 locus in MISTRG6 mice enhanced HSPC engraftment, enabling autologous modeling of tumor–immune dynamics directly from a patient’s BM aspirate. The autologous tumors recreated key elements of the human TME, including activation of both innate and adaptive immunity, thereby providing a powerful platform for preclinical drug testing [204]. Nevertheless, detailed phenotypic maturation of these NK cells (e.g., expression of KIR, CD57) was not deeply characterized in that context.

NK cells’ development and function have been much better studied in MISTRG-6-15 (knock-in of human IL-6 and IL-15) humanized mice. In this model, Sungur et al. showed rapid NK cell expansion and, upon HIV-1 infection, strong degranulation, cytotoxicity (against the K562 cell line), and pro-inflammatory cytokine production in non-lymphoid tissues [169]. In these mice, NK cells in lymphoid organs showed more immature phenotypes (lower CD16A) and a reduced function, mirroring some features of human tissue-resident NK cells. Importantly, depletion of NK cells (via anti-NKp46) in vivo led to significantly higher HIV-1 replication, demonstrating that these NK cells exert functional antiviral control in vivo [169].

Importantly, “double-humanized” platforms, such as human-BM–Liver–Thymus (hu-BLT) mice, use models combining HSPC engraftment with tissue-specific human grafts, enable the study of NK cell trafficking and infiltration into organ-specific niches. Experimental studies in hu-BLT systems show that human NK cells (transferred or endogenously reconstituted) can reach xenografted tumors, produce IFN-γ, mediate cytotoxicity, and in some cases, restore or potentiate endogenous NK cell function. An additional hu-BLT study has reported NK cell tumor infiltration and functional activation (IFN-γ, cytotoxicity) [205,206]. However, these platforms do not yet fully recapitulate the human vasculature, chemokine gradients, and stromal architecture, which should be considered when interpreting tissue-level immune behaviors [165]. Taken together, the available evidence demonstrates that cytokine-enhanced humanized mouse strains substantially overcome the key limitations of first-generation platforms. By providing sustained human cytokine support, these models achieve markedly higher levels of human NK cell reconstitution, promote the emergence of more mature and functionally competent NK subsets (including CD56^dim^/CD16^bright^, KIR^+^, perforin^+^/granzyme^+^ populations), and support robust cytotoxic and cytokine-producing activity in vivo. Importantly, they enable physiologically relevant NK cell trafficking, higher tumor infiltration, and therapeutic responsiveness within human tumor xenograft settings, features largely absent from classical models. Nevertheless, even in cytokine-optimized platforms, the licensing and education of NK cells are constrained by incomplete humanization of the MHC class I landscape. Mouse models expressing KIR2DL2 receptors together with their HLA-Cw3 ligand have demonstrated that HLA class I influences the KIR repertoire and education [207]. In humanized NSG mice, for example, KIR-mediated NK education critically depends on the presence of matching HLA ligands in trans, and mixed-HLA reconstitution can abrogate this education [208]. Although emerging transgenic and knock-in strategies offer promising routes, full recreation of a physiological KIR-HLA class I axis—including classical (HLA-A, -B, -C) and nonclassical (HLA-E/F) class I molecules—remains largely unexplored. A comprehensive summary of the mouse models described in this review is provided in Table 1.

## 6. Conclusions and Future Perspectives

Collectively, the studies described and discussed in this review position cytokine-enhanced humanized mice as powerful and increasingly indispensable systems for dissecting the human NK cell biology and evaluating NK-mediated anti-cancer immunotherapies. Yet, their continued refinement, particularly with respect to HLA class I humanization, the tissue-specific immune architecture, and preservation of NK cell education, will be essential to fully unlock their predictive and translational potential.

In addition to that, the combination of multi-omics analytic platforms will speed up the optimization of the models. Historically, in vivo studies of human NK cells were largely limited to measurements of the frequency, surface phenotype, cytotoxicity, and cytokine production, providing little insight into their temporal dynamics or tissue-specific behavior. The integration of longitudinal monitoring with single-cell multi-omics, intravital imaging, and in vivo tracking (PET/SPECT) has transformed this landscape, offering a far more detailed view of NK cell biology in both the systemic circulation and TME. For example, in a humanized mouse model with IL-15 knock-in, researchers combined scRNA-seq with repeated functional assays to track NK cells over time in hepatocellular carcinoma xenografts [139]. This approach identified transient subpopulations exhibiting activation markers early after infusion and, later, upregulation of exhaustion-associated genes such as PDCD1 and TIGIT, which would have been missed by conventional analyses. Functional readouts correlated with transcriptional states, revealing a progressive loss of cytotoxicity in specific tissue compartments and highlighting the dynamic interplay between NK activation and suppression in vivo. Similarly, longitudinal studies in MISTRG-6-15 humanized mice demonstrated that NK cells initially expand and display high cytotoxic and metabolic activity, but over weeks, some subsets undergo transcriptional and metabolic reprogramming toward reduced oxidative phosphorylation and glycolytic capacity [209]. By integrating single-cell transcriptomics with functional assays, these studies revealed how metabolic exhaustion develops in vivo, insights inaccessible in static models. Regarding the possibility of tracking NK cells, recent studies highlighted the importance of integrating PET scanning into preclinical investigation. PET imaging using ^89^Zr-oxine-labeled human NK cells has enabled whole-body tracking of adoptively transferred NK cells over time: in a model of HER2-positive breast cancer, ^89^Zr-labeled NK cells were visualized by PET/CT for up to seven days, revealing their accumulation in the liver and spleen, and enhanced tumor infiltration when co-administered with trastuzumab [210]. Similarly, in non-human primates, serial PET/CT of ^89^Zr-oxine-labeled NK cells showed initial localization in the lungs followed by migration to the liver and spleen, demonstrating the feasibility of quantitatively tracking NK cell biodistribution in vivo [211]. Together, these technologies transcend traditional endpoint measurements by providing time-resolved, spatially resolved, and molecularly detailed insights into NK cell trafficking, activation, dysfunction, and therapeutic responsiveness, insights that were largely inaccessible with earlier in vitro or in vivo assays.

To conclude, recent innovations have led to significant advances in humanized models, imaging technologies, and analytical pipelines, thereby enhancing both the resolution and physiological relevance. Building on this foundation, cytokine-enhanced humanized mouse models may more accurately recapitulate human NK cell biology. Reconstruction of tissue-specific immune niches and optimized cytokine support are expected to promote NK cell education, survival, and effector function. The incorporation of human stromal and endothelial components into three-dimensional scaffolds can generate microenvironments that more closely resemble the in vivo conditions. The integration of multi-omics analyses with functional assays and advanced imaging techniques, including intravital microscopy and PET/CT of labeled NK cells, will enable the dynamic and spatially resolved assessment of NK cell activation, exhaustion, and interactions within the tumor microenvironment. Collectively, these strategies are anticipated to broaden the understanding of human NK cell behavior, enhance the translational relevance of preclinical studies, and support the rational development and prioritization of NK cell-based immunotherapies, thereby reducing the risk of clinical setbacks.

## Figures and Tables

**Table 1:** Evolution of humanized mouse models for NK cell research.

	Mouse Model	Humanization Strategy	NK Cell Reconstitution	NK Subset Distribution	Functional Maturation and Activity	Tumor Infiltration/In Vivo Relevance
**Early immunodeficient models**	**NOD/SCID**	T^−^ B^−^ (residual NK activity)	Very low	Not physiologic	Short-term cytotoxicity	Limited, transient
	**NSG**	T^−^ B^−^ NK^−^ (IL2Rγ^−/−^)	Very low	Infused NK phenotype only	Limited survival and function	Weak tumor control
	**NOG**	T^−^ B^−^ NK^−^ (IL2Rγ^−/−^)	Very low	Infused NK phenotype only	Poor	Limited
**First-generation humanized models**	**NSG-SGM3**	hSCF, hGM-CSF, hIL-3 transgenic	Low–moderate	Predominantly CD56^bright^	Partial maturation	Limited
	**MISTRG**	hM-CSF, hIL-3/GM-CSF, hTPO, hSIRPα knock-in	Moderate–high	More mature NK cells	Improved cytotoxicity	Improved vs. NSG
	**SRG-15**	hIL-15 knock-in + hSIRPα	High	Balanced CD56^bright^/CD56^dim^	Robust ADCC	Efficient tumor infiltration
	**NSG-IL15**	Constitutive or knock-in hIL-15	High	Both major subsets present	High perforin/granzyme expression	Strong tumor control
**Cytokine-enhanced** **second generation models**	**NSG-hIL7-hIL15**	Human IL-7 + IL-15 knock-in/transgenic	Very high	Expanded NK compartment	Strong cytotoxicity	High infiltration
	**MISTRG6**	MISTRG + human IL-6 knock-in	High	Functionally competent NK cells	Supports immune–tumor crosstalk	Autologous tumor control
	**MISTRG6-15**	MISTRG6 + human IL-15 knock-in	High	Tissue-heterogeneous NK cells	Models chronic activation and exhaustion	Tissue-dependent responses
**Double-humanized next generation models**	**hu-BLT**	HSPCs + human liver and thymus grafts	Moderate	Circulating and tissue NK cells	IFN-γ production, cytotoxicity	Tumor infiltration reported
	**Autologous humanized host + matched PDX**	Pt-derived BM CD34^+^ HSPCs + matched PDX	Moderate–high	Patient-specific NK composition	Activates innate immunity in autologous TME	Patient-matched tumor–immune interactions

## Data Availability

No new data were created in this study. The data presented in this study are available at references included in the bibliography.

## Abbreviations

The following abbreviations are used in this manuscript:

ADCC	Antibody-Dependent Cellular Cytotoxicity
ALL	Acute Lymphoblastic Leukemia
AML	Acute Myeloid Leukemia
ANKETs	Antibody-based NK cell Engager Therapeutics
BBB	Blood–Brain Barrier
B-NHL	B-cell Non-Hodgkin Lymphoma
BiKEs	Bispecific NK cell Engagers
BM	Bone Marrow
CAR-NK	Chimeric Antigen Receptor-Natural Killer
CIML	Cytokine Induced Memory-Like
CRS	Cytokine Release Syndrome
DC	Dendritic Cell
GBM	Glioblastoma Multiforme
GvHD	Graft-versus-Host Disease
HD	Healthy Donor
hESCs	Human Embryonic Stem Cells
HSC	Hematopoietic Stem Cell
HSPCs	Hematopoietic Stem and Progenitor Cells
hu-BLT	Human BM Liver Thymus
IL-2	Interleukin-2
IL-2v	IL-2 variant
ILC	Innate Lymphoid Cell
iPSC	Induced Pluripotent Stem Cell
KIR	Killer Ig-like Receptor
mAbs	Monoclonal Antibodies
MISTRG	M-CSF^h/h^ IL-3/GM-CSF^h/h^ SIRPα^h/h^ TPO^h/h^ RAG2^−/−^ IL2Rg^−/−^
NCR	Natural Cytotoxicity Receptor
NK	Natural Killer
NKCEs	NK Cell Engagers
NOD/SCID	Non-Obese Diabetic/Severe Combined Immunodeficient
NOG	NOD/Shi-scid/IL2Rγ^null^
NSG	NOD/SCID/γc^null^
NSG-IL15	Transgenic for Human IL-15
NSG-SGM3	NOD-scid IL2rγ^−/−^ Transgenic for Human SCF, GM-CSF, and IL-3
PB	Peripheral Blood
PDX	Patient Derived Xenograft
sc RNA-seq	Single-cell RNA Sequencing
scFv	Single-chain variable Fragment
SRG-15	RAG2^−/−^ IL2Rg^−/−^ Transgenic for Human IL-15
TAAs	Tumor-Associated Antigens
TAMs	Tumor-Associated Macrophages
TCEs	T Cell Engagers
TME	Tumor Microenvironment
Tregs	Regulatory T cells
TriKEs	Trispecific NK Cell Engagers
UBC	Umbilical Cord Blood
